# Supplementary material for: Mycelium-mediated transfer of water and nutrients stimulates bacterial activity in dry and oligotrophic environments
Source: Nat Commun. 2017 Jun 7;8:15472. doi: 10.1038/ncomms15472 (PMC5467244; doi:10.1038/ncomms15472)
Supplement: Supplementary Information — Supplementary Figures and Supplementary Table [file ncomms15472-s1.pdf]

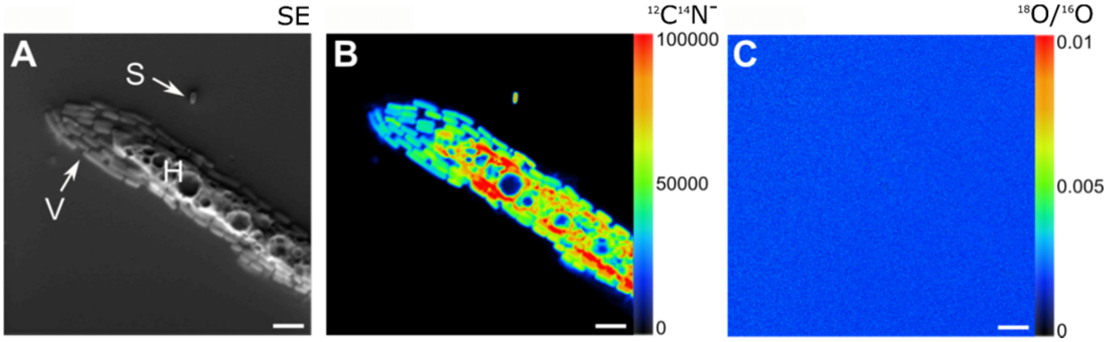

Supplementary Figure 1: NanoSIMS images of *P. ultimum* hyphae (H), *B. subtilis* spores (S) and vegetative cells (V) identified in a secondary electron and total biomass ($^{12}C^{14}N^{-}$) image (A, B) of a non-labeled sample. The ratio images of $^{18}O/^{16}O$ show the natural abundance of ^{18}O in the biomass of *P. ultimum* and *B. subtilis* (C). The color scale indicates the intensities of $^{12}C^{14}N^{-}$ (B) and enrichment in ^{18}O (C) with warmer colors representing higher secondary ion counts and cooler colors representing lower values. Images represent a field of analysis corresponding to a sample area of 40×40 µm. Scale bars, 4 µm.

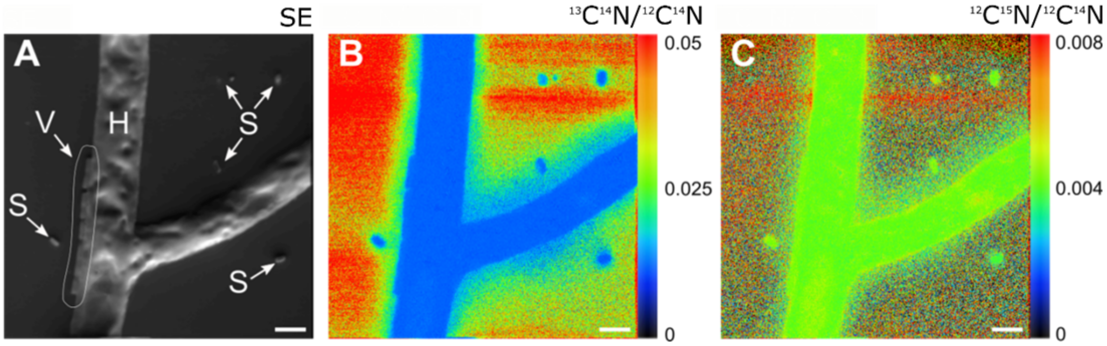

Supplementary Figure 2: NanoSIMS images of *P. ultimum* hyphae (H), *B. subtilis* spores (S) and vegetative cells (V) identified in a secondary electron image (A) of a non-labeled sample. The ratio images of $^{13}C^{14}N/^{12}C^{14}N$ (B) and $^{12}C^{15}N/^{12}C^{14}N$ (C) show the natural abundance of ^{13}C and ^{15}N in the biomass of *P. ultimum* and *B. subtilis*. The color scale indicates the intensities of $^{13}C^{14}N/^{12}C^{14}N$ (B) and $^{12}C^{15}N/^{12}C^{14}N$ (C) enrichments with warmer colors representing higher enrichment levels and cooler colors representing lower values. Images represent a field of analysis corresponding to a sample area of 40×40 µm. Scale bars, 4 µm.

Supplementary Table 1: Number (#) of individual fields and replicate wafers analysed with NanoSIMS in ^{18}O and $^{13}C^{15}N$ labeling experiments and ^{16}O and $^{12}C^{14}N$ control experiments as well as numbers of vegetative cells and spores used to calculate APE shown in Fig. 6.

**Sample**	**# Fields**	**# Wafers**	**# Vegetative cells**	**# Spores**
^{18}O	3	2	20	23
^{16}O	2	2	106	3
$^{13}C/^{15}N$	2	1	57	11
$^{12}C/^{14}N$	3	2	104	7
